# Unveiling Symmetry Through Orthodontic-Surgical Management of Unilateral Mandibular Condylar Agenesis: A Rare Case Report

**DOI:** 10.7759/cureus.59294

**Published:** 2024-04-29

**Authors:** Kushal Taori, Priyanka Niranjane, Ranjit Kamble, Shriya Murarka, Radhika Tayshetye

**Affiliations:** 1 Department of Orthodontics, Sharad Pawar Dental College, Wardha, IND; 2 Department of Oral and Maxillofacial Surgery, Sharad Pawar Dental College, Wardha, IND

**Keywords:** mandible, condyle, temporomandibular joint, condylar aplasia, unilateral, condylar agenesis

## Abstract

This is a rare clinical case report of a 19-year-old male patient reported in the Department of Orthodontics and Dentofacial Orthodontics, Sharad Pawar Dental College, Sawangi (Meghe), Wardha, Maharashtra, with chief complaint of asymmetry present on the lower left side of the face and forwardly placed upper front teeth. The asymmetry was due to the congenital complete absence of the left condyle and skeletal cant in the maxilla and functional occlusal plane. The true agenesis of the condyle is an extremely rare condition that requires proper diagnosis and interdisciplinary management. In this case, there was left-sided condylar agenesis with no other skeletal anomaly present, which was treated by pre-surgical orthodontics for decompensation and surgical correction of skeletal cant followed by Ramal distraction osteogenesis and advancement genioplasty. A condylar stock metal implant was placed on the left side for functional rehabilitation. The patient expressed satisfaction with the aesthetic and functional improvements, highlighting the effectiveness of the integrated orthodontic-surgical approach.

## Introduction

The temporomandibular joint (TMJ) is indeed a unique structure in the body, crucial for the functionality of the jaw and surrounding structures. It comprises the mandibular condyle and the articular eminence of the temporal bone, with a fibrous disc between them, dividing the joint into two compartments [1,2]. This arrangement allows for both rotational and translatory movements, making TMJ a ginglymus-diarthrodial joint. One notable feature of the TMJ is its bilateral diarthrodial nature, meaning both the left and right sides must function together for proper movement and alignment [2,3]. This bilateral functionality is crucial for activities such as chewing, speaking, and swallowing. Various factors can lead to unilateral or bilateral growth disturbances of the mandibular condyle and its associated structures. Apart from congenital and accidental factors, conditions like TMJ arthritis, disc displacement, or chronic inflammation like rheumatoid arthritis, malocclusion of upper and lower jaws can lead to asymmetric growth. Idiopathic causes like improper orthodontic correction can lead to growth disturbances in the mandibular condyle. These disturbances can manifest in different ways, affecting the size, shape, or functionality of the TMJ. Some growth disturbances may occur during prenatal development, leading to conditions such as aplasia or hypoplasia of the mandibular condyle and its soft tissues. These abnormalities may arise late in the first trimester and can result in significant facial abnormalities [2,4]. On the other hand, disturbances during the normal growth period can lead to condylar hyperplasia, characterized by abnormal local growth stimulation. This condition can cause overgrowth of the mandibular condyle, impacting the function and aesthetics of the TMJ and surrounding structures [2,5]. Understanding the various causes and consequences of growth disturbances in the mandibular condyle is essential for diagnosing and managing conditions related to TMJ dysfunction and facial abnormalities.

Aplasia of the mandibular condyle is a rare anomaly, having an incidence of one in 5,600 births [6]. It occurs more in males and the incidence of unilateral aplasia/agenesis is more than the bilateral aplasia. Any disturbance of the condylar cartilage that decreases its growth activity will result in underdevelopment of the mandible [7]. Aplasia and hypoplasia of the mandibular condyle may be associated with the microtia, part of the temporal bone, and the absence of the entire ramus. Unilateral aplasia and hypoplasia of the mandibular condyle lead to the underdevelopment of the mandible, resulting in a lack of symmetrical growth of the mandible, latrognathy, micrognathia characterized by bird face, and a markedly short mandible unilaterally on the affected side [8].

## Case presentation

**Figure 1:**
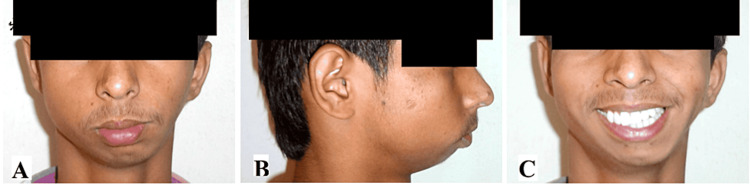
Pre-treatment extraoral photographs (A) Frontal view, (B) profile view, (C) frontal-smiling view

A 19-year-old male patient reported to the Department of Orthodontics and Dentofacial Orthopedics, Sharad Pawar Dental College, Sawangi (Meghe), Wardha, MH, India with a chief complaint of poor esthetics due to forwardly placed upper front teeth and asymmetry in the lower third of the face. There was no past medical/dental history, no history of deleterious habits, and no trauma in the facial region. On extra-oral examination, there was asymmetry on the lower third of the face with the lower midline not matching the facial midline and shifted towards the left side of the face. Profile was convex with posterior divergence, mesocephalic head form, and mesoprosopic facial form. The perpendicular distance from the line passing through the nasal base to the left oral commissure was reduced compared to the right side (Figures 1A-1C).

**Figure 2:**
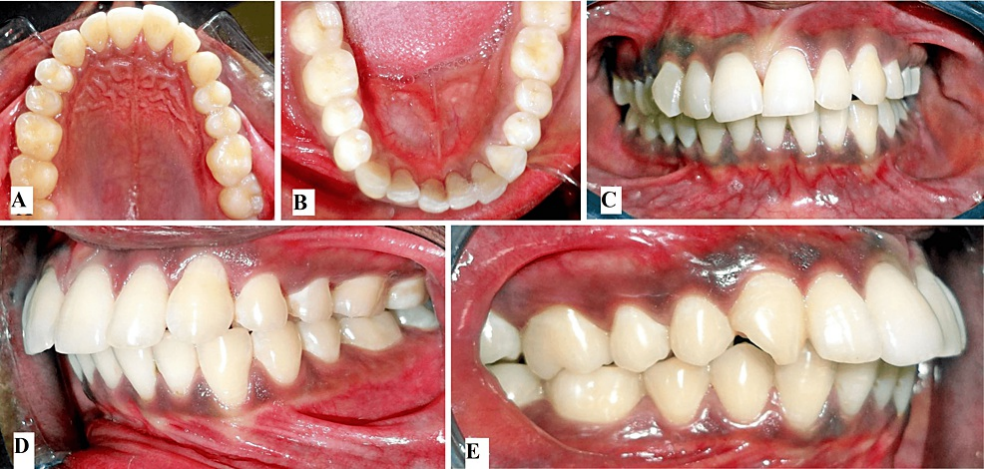
Pre-treatment intra-oral photographs (A) Upper intraoral occlusal view, (B) lower intraoral occlusal view, (C) intraoral frontal view showing occlusal cant, (D) intraoral left side showing reduced alveolar height, (E) intraoral right side

Intraoral examination revealed cant in the functional occlusal plane, lower midline shifted towards left side by 3.5mm, proclined upper incisors, mesially rotated upper right canine, compensatory lingual tilting of left mandibular posteriors, and mild lower anterior crowding. Sulcular height at the maxillary premolar region was reduced on the left side compared with the right side, this represents canting in the maxillary and mandibular arch towards the left side (Figures 2A-2E).

**Figure 3:**
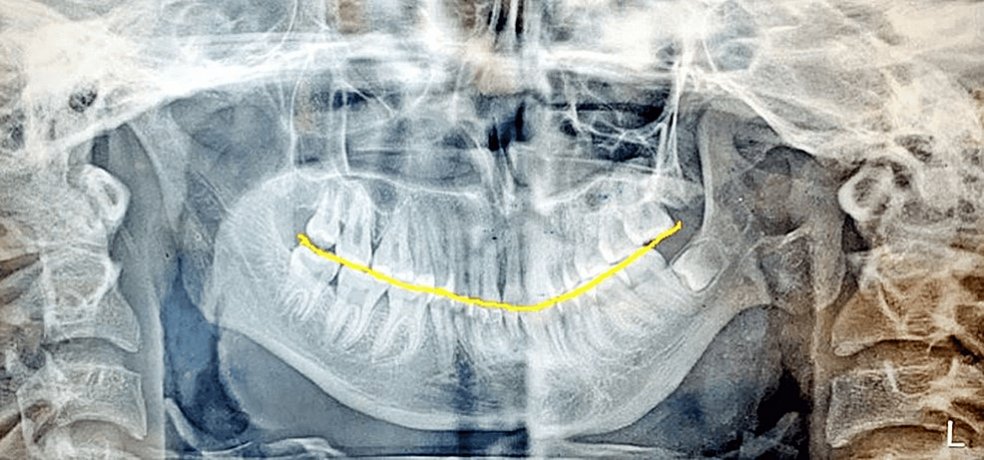
Orthopantomogram shows deep antegonial notch on the ipsilateral side and complete absence of unilateral left condyle

**Figure 4:**
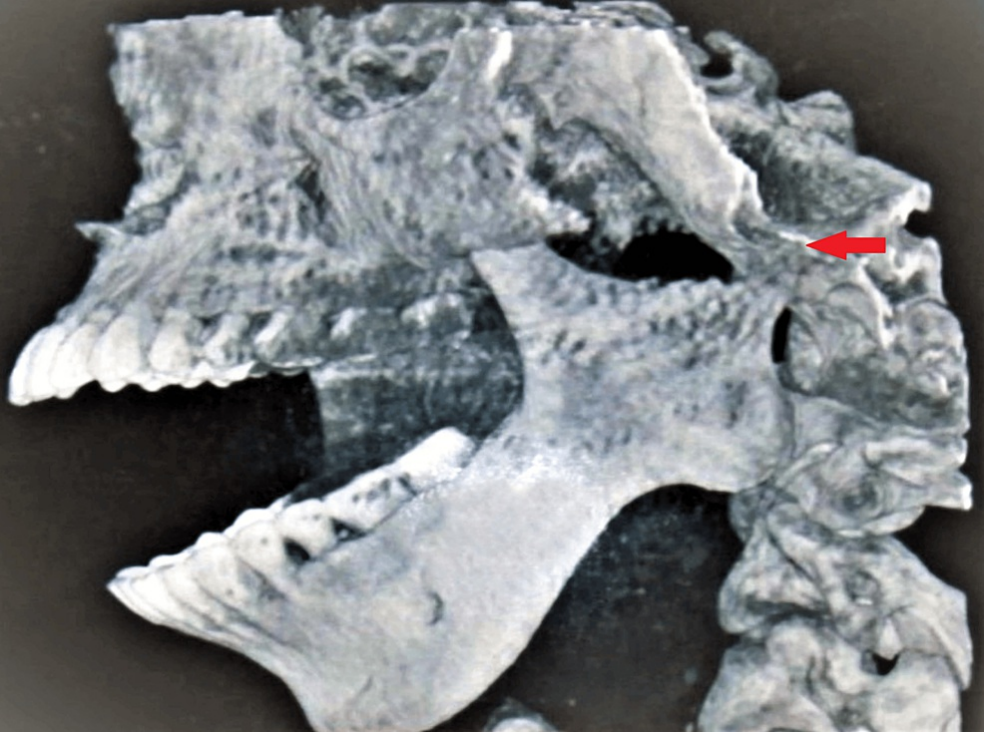
CBCT image shows complete absence of left condyle till the level of sigmoid notch

**Figure 5:**
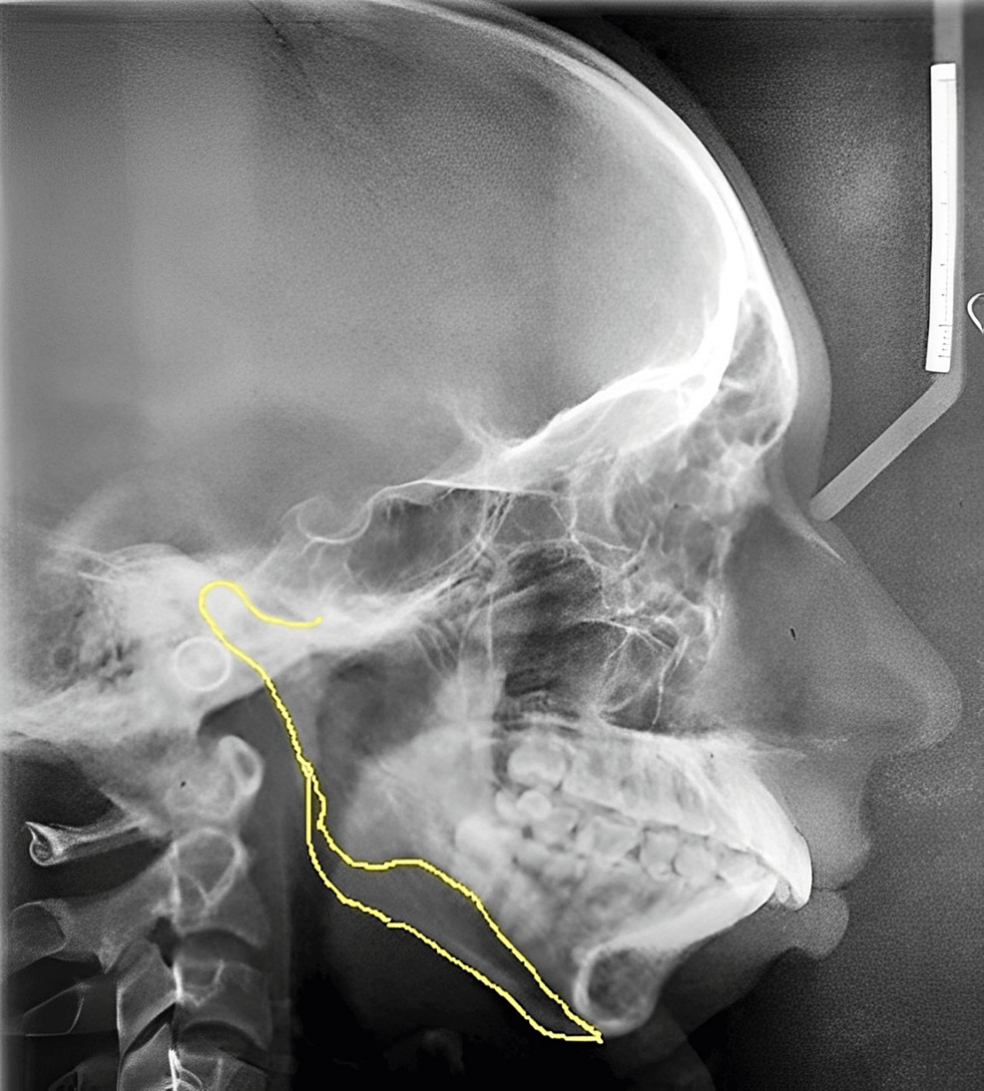
Pre-treatment lateral cephalogram

On radiographic examination, an orthopantomogram revealed a complete absence of condyle on the left side with a prominent antegonial notch on the left lower border of the mandible. Ramus is terminated relatively flat at the level of a sigmoid notch on the left side (Figure 3). CBCT confirmed the complete absence of the left side of the mandibular condyle which led to a deviation of the mandible on the ipsilateral side (Figure 4). Lateral cephalograms showed class II skeletal bases with orthognathic maxilla and retrognathic mandible and increased lower facial submental angle (Figure 5).

After correlation of clinical and radiographic analysis, the case was diagnosed as Skeletal Class II malocclusion with Angle’s Dentoalveolar Class II, Division 1, subdivision malocclusion with Condylar agenesis on the left side, adaptive changes in maxillary base and convex profile.

Treatment objectives

The primary objective was to correct the facial asymmetry and functional disability. This was achieved by decompensation of the dental arches, skeletal correction of the cant in the maxilla and mandible, management of the condylar agenesis on the left side, correction of the convex profile for aesthetic rehabilitation, and correction of the lower facial submental angle.

Treatment plan

In the comprehensive treatment plan for correcting skeletal discrepancies and achieving optimal facial harmony, a structured approach was employed, encompassing three distinct phases. Phase I involved pre-surgical orthodontics aimed at leveling and aligning the dentition utilizing fixed orthodontic therapy. This phase also involved decompensating the maxillary and mandibular arches, preparing them for subsequent surgical intervention. Phase II constituted the surgical phase, with Step 1 entailing the meticulous placement of a condylar graft, LeFort-I osteotomy, and mandibular Ramal distraction osteogenesis, specifically targeting the left side for correction. Step 2 involved advancement genioplasty to harmonize the facial profile further. Finally, Phase III encompassed post-surgical orthodontics, focusing on refining the occlusion, achieving proper dental alignment, and ensuring the stability of the surgical outcomes. This structured approach ensured the comprehensive correction of skeletal discrepancies, culminating in a harmonious and functional dentofacial relationship.

Treatment progress

**Figure 6:**
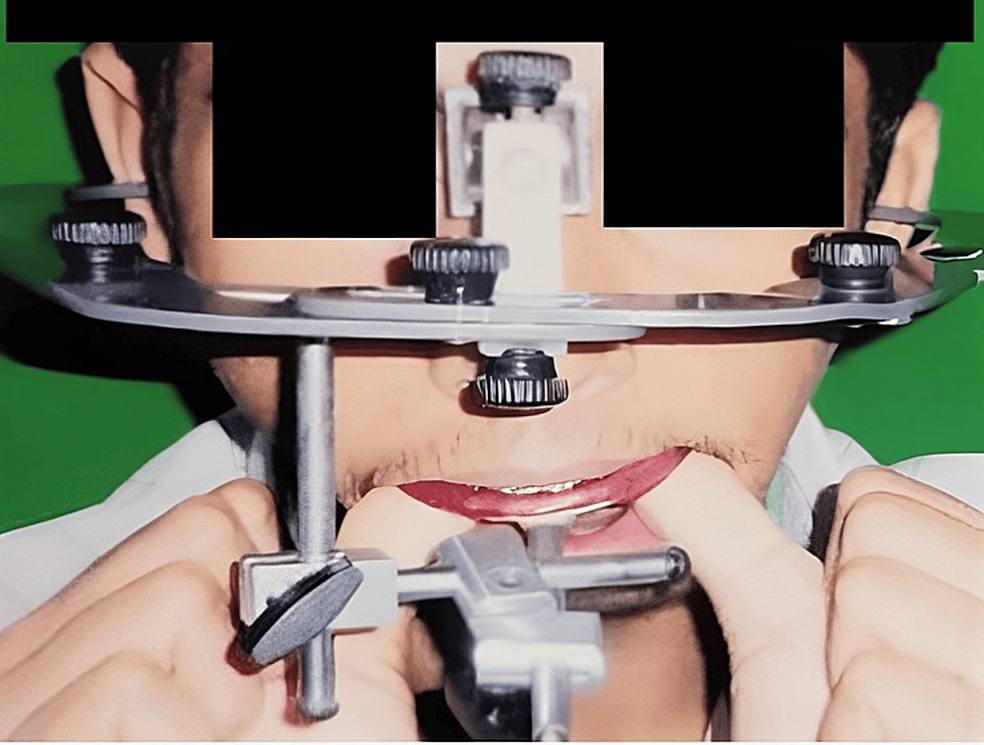
Facebow Transfer

Maxillary and mandibular arches were bonded with a 0.022-inch pre-adjusted edgewise appliance. Sequential use of Ni-Ti and stainless-steel wires were used for initial leveling and alignment of the arches starting from round Ni-Ti of 0.016-inch diameter followed by rectangular stainless-steel wires. The alignment of both the arches was done till 0.021” x 0.025” stainless steel rectangular wire. Post-alignment records were taken, and the case was discussed with an oral surgeon. Face bow transfer was done for mock surgery (Figure 6).

**Figure 7:**
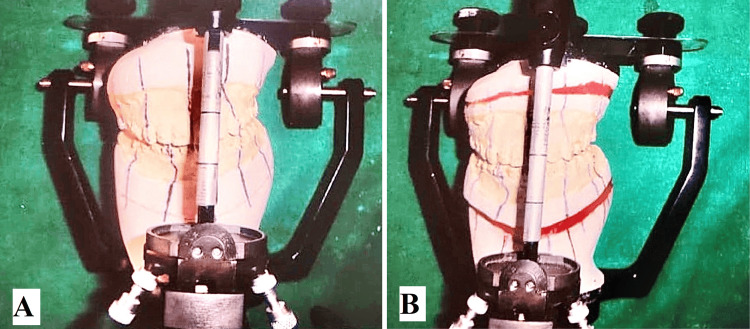
Mock surgery models articulated on articulator after facebow transfer (A) Before mock surgery model, (B) after mock surgery model

Vertical distraction on the left side was planned with the maxilla and Ramal distraction on the mandible for occlusal cant and asymmetry correction on the mock surgery model which was augmented by advancement genioplasty (Figures 7A, 7B).

**Figure 8:**
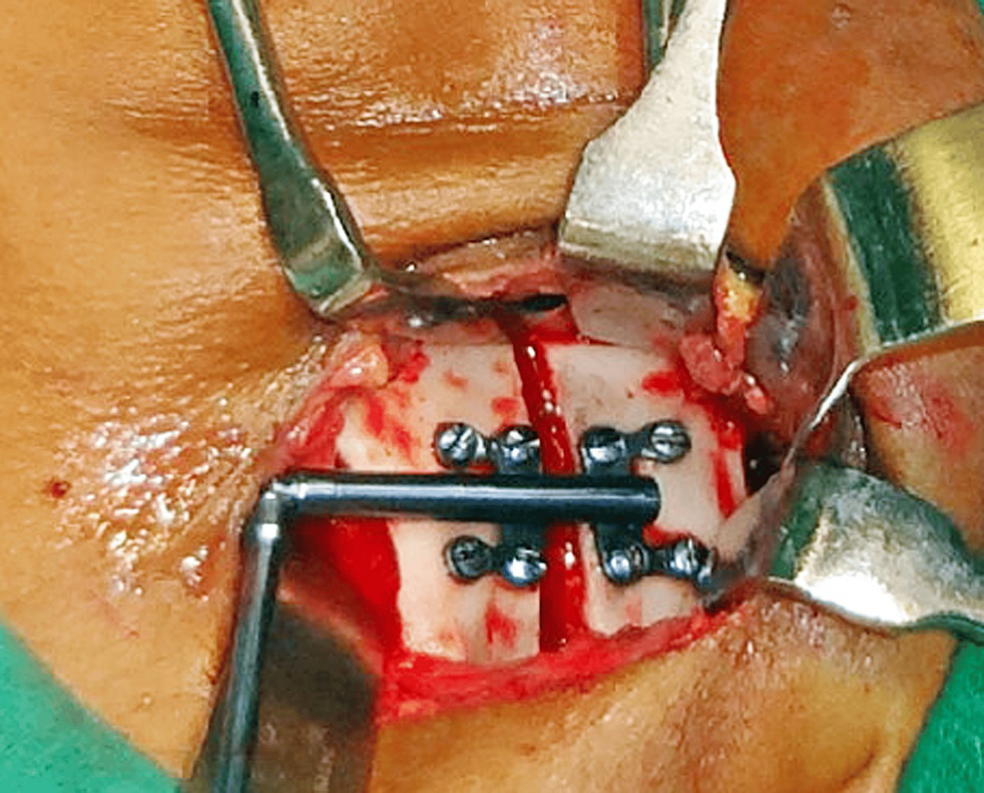
Placement of unidirectional Ramal Distractor for Distraction Osteogenesis

**Figure 9:**
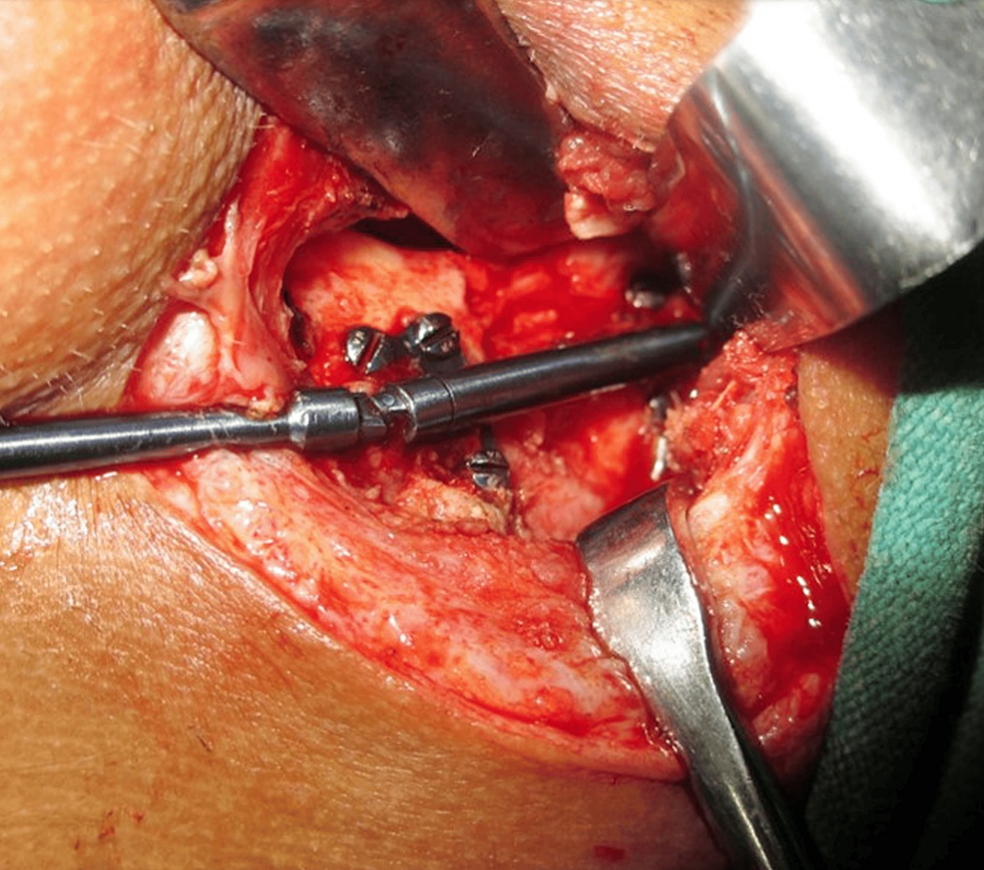
Post-distraction osteogenesis photograph shows 25mm bone formation after consolidation phase.

**Figure 10:**
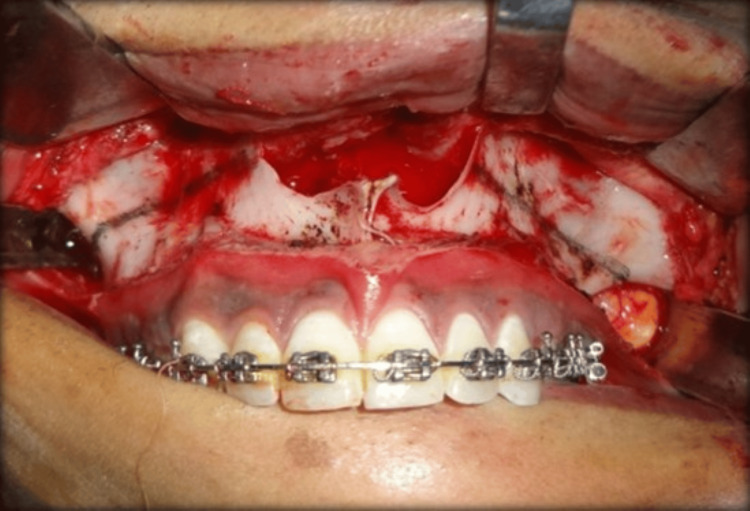
LeFort-I Osteotomy

The patient was referred to the Department of Oral and Maxillofacial Surgery for further surgical management. Inter-maxillary fixation was done which was followed by Ramal osteotomy and placement of a unidirectional Ramal distractor for distraction osteogenesis of the left side of the mandible (Figure 8). A total of 25 mm distraction was achieved on the left side of the mandible at a rate of 1 mm per day (Figure 9). LeFort I osteotomy was done for maxillary cant correction. (Figure 10). A condylar stock implant was placed in the region of the left mandibular condyle for functional rehabilitation of the joint.

Treatment outcome

**Figure 11:**
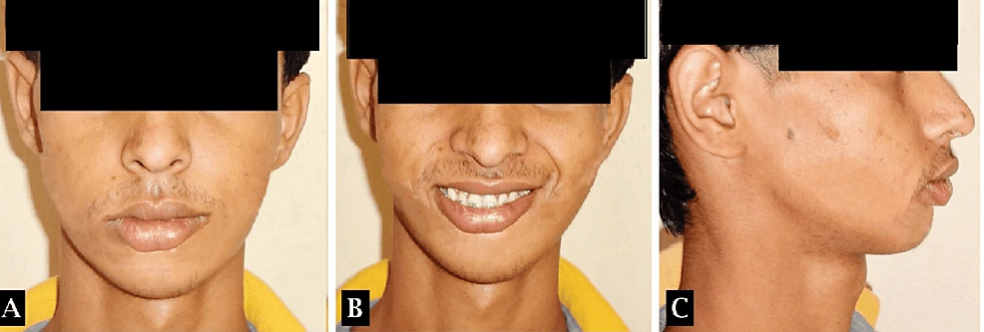
Post-treatment extraoral photographs (A) Frontal view, (B) frontal smiling view, (C) profile view

**Figure 12:**
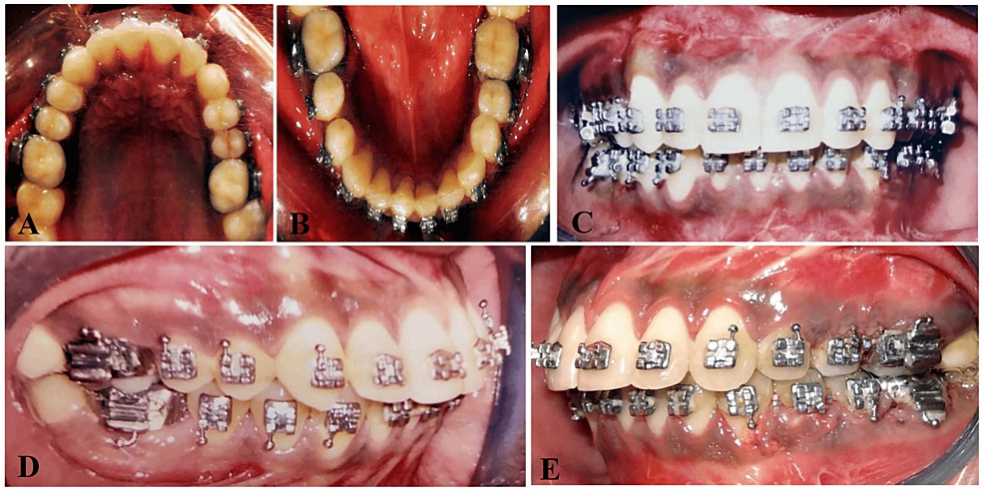
Post-treatment intraoral photographs (A) Intraoral maxillary occlusal view, (B) intraoral mandibular occlusal view, (C) intraoral frontal view showing correction of occlusal cant, (D) intraoral right side occlusal view, (E) intraoral left side occlusal view showing increased alveolar height

**Figure 13:**
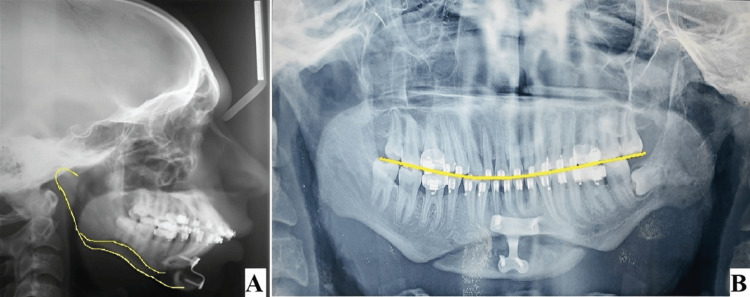
Post-treatment lateral cephalogram and orthopantomogram (A) Post-treatment lateral cephalogram shows improvement in the profile, (B) post-treatment orthopantomogram shows correction in the occlusal cant

**Figure 14:**
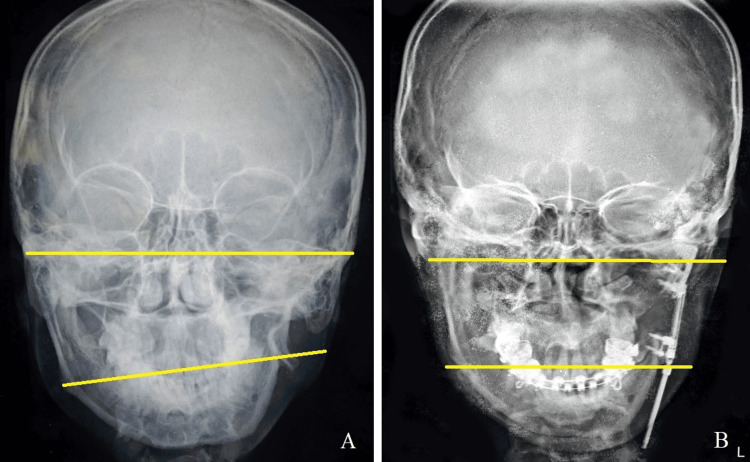
Pre-treatment and post-treatment comparison of postero-anterior cephalogram (A) Pre-treatment cephalogram, (B) post-treatment cephalogram

Following a LeFort I osteotomy and Ramal distraction osteogenesis performed on the left side of the maxilla and mandible respectively, corrections were made to a maxillary skeletal cant by 4mm and an occlusal cant by 3.5mm. Ramal distraction achieved by 25mm on the left side, complemented by advancement genioplasty to address skeletal asymmetry and a class II correction on the left side (Figures 11-14). Orthodontic intervention successfully aligned the arches and settled the occlusion, utilizing a 0.021” x 0.025” stainless steel rectangular wire. The patient expressed satisfaction with the treatment outcomes.

## Discussion

Aplasia of the mandibular condyle is a rare condition, often associated with syndromes like hemifacial microsomia, Goldenhar syndrome, and Treacher-Collins syndrome, among others. Additionally, Proteus syndrome, Morquio syndrome, and auriculocondylar syndrome may also present condylar malformations [9-11]. While these syndromes typically entail various facial and skeletal anomalies alongside condylar aplasia, isolated cases devoid of other developmental abnormalities are exceedingly rare [9,10].

Mechanical trauma during active growth is the primary cause of condyle alterations post-birth, although inflammation in the TMJ area, rheumatoid arthritis, radiotherapy, and deficiencies in parathyroid hormone-related protein can also lead to condylar malformation [12].

Condylar hypoplasia, ranging from minimal to near-complete absence, can result from abnormal growth and development. During prenatal and postnatal condylar growth, the primitive joint within Meckel’s cartilage briefly functions as a jaw joint before the formation of the definitive TMJ. This developmental process involves synchronized movements of the incudomalleal joint and the developing TMJ, regulated by muscles innervated by the mandibular division of the trigeminal nerve. The TMJ evolves from initially separate temporal and condylar blastemata that gradually converge. Unilateral or bilateral aplasia of the condyle likely occurs before the 10th week post-conception when condylar cartilage begins to develop [13]. The typical treatment approach involves surgical procedures such as unilateral ramal osteotomy and distraction osteogenesis to either shorten the unaffected side of the mandible or lengthen the affected side, as illustrated in the aforementioned case. Prior to surgery, orthodontic therapy is provided to prepare and enhance outcomes. In younger patients with developing jaws, orthopedic interventions utilizing functional appliances are often utilized to correct deformities or prevent their exacerbation as they mature. Once growth has ceased, correcting skeletal deformities typically necessitates double jaw surgery, genioplasty, or unilateral mandibular augmentation [14]. In our case, the affected temporo-mandibular joint on the left side due to agenesis was replaced by a condylar stock implant. This was done because costochondral grafts show unpredictable growth at the reconstruction site which leads to further abnormal development of the mandible [15].

## Conclusions

The successful treatment of the presented case underscores the importance of an interdisciplinary approach in addressing complex craniofacial anomalies. By integrating orthodontic treatment planning and surgical interventions tailored to the patient's specific needs, significant improvements in facial symmetry, occlusal alignment, and functional rehabilitation were achieved. The comprehensive management strategy not only corrected skeletal discrepancies and malocclusion but also addressed aesthetic concerns, resulting in enhanced patient satisfaction. This case serves as a testament to the efficacy of collaborative efforts between orthodontic, oral, and maxillofacial surgical teams in achieving favorable outcomes for patients with challenging craniofacial conditions.
